# Characterization of *N*-Acylhomoserine Lactones Produced by Bacteria Isolated from Industrial Cooling Water Systems

**DOI:** 10.3390/s16010044

**Published:** 2015-12-30

**Authors:** Noriya Okutsu, Tomohiro Morohoshi, Xiaonan Xie, Norihiro Kato, Tsukasa Ikeda

**Affiliations:** 1 Department of Material and Environmental Chemistry, Graduate School of Engineering, Utsunomiya University, 7-1-2 Yoto, Utsunomiya, Tochigi 321-8585, Japan; noriya.okutsu@kurita.co.jp (N.O.); katon@cc.utsunomiya-u.ac.jp (N.K.); tikeda@cc.utsunomiya-u.ac.jp (T.I.); 2 Center for Bioscience Research and Education, Utsunomiya University, 350 Mine-machi, Utsunomiya, Tochigi 321-8505, Japan; xie@cc.utsunomiya-u.ac.jp

**Keywords:** quorum sensing, *N*-acylhomoserine lactone, industrial cooling water system, *Bosea massiliensis*, *Lysobacter* sp.

## Abstract

The cooling water systems are used to remove heat generated in the various industries. Biofouling of the cooling water systems causes blocking of condenser pipes and the heat exchanger tubes. In many Gram-negative bacteria, *N*-acylhomoserine lactone (AHL) are used as quorum-sensing signal molecule and associated with biofilm formation. To investigate the relationship between quorum sensing and biofouling in the cooling water system, we isolated a total of 192 bacterial strains from the five cooling water systems, and screened for AHL production. Seven isolates stimulated AHL-mediated purple pigment production in AHL reporter strain *Chromobacterium violaceum* CV026 or VIR07. Based on their 16S rRNA gene sequences, AHL-producing isolates were assigned to *Aeromonas hydrophila*, *Lysobacter* sp., *Methylobacterium oryzae*, and *Bosea massiliensis*. To the best of our knowledge, *B. massiliensis* and *Lysobacter* sp. have not been reported as AHL-producing species in the previous researches. AHLs extracted from the culture supernatants of *B. massiliensis* and *Lysobacter* sp. were identified by liquid chromatography-mass spectrometry. AHLs produced by *B. massiliensis* were assigned as *N*-hexanoyl-l-homoserine lactone (C6-HSL), *N*-(3-oxohexanoyl)-l-homoserine lactone (3-oxo-C6-HSL), and *N*-(3-oxooctanoyl)-l-homoserine lactone (3-oxo-C8-HSL). AHLs produced by *Lysobacter* sp. were assigned as *N*-decanoyl-l-homoserine lactone (C10-HSL) and *N*-(3-oxodecanoyl)-l-homoserine lactone (3-oxo-C10-HSL). This is the first report of identification of AHLs produced by *B. massiliensis* and *Lysobacter* sp. isolated from the cooling water system.

## 1. Introduction

Quorum sensing is one of the cell-cell communication systems depending on cell population density [1]. Quorum-sensing signaling is mediated by an autoinducer, which is a self-produced signal molecule [1]. In many Gram-negative bacteria, several kinds of *N*-acylhomoserine lactone (AHL) have been identified as autoinducer signal compounds involved in this mechanism [2]. The substrates in AHL synthesis are *S*-adenosyl methionine (SAM) and acyl-acyl carrier proteins. AHLs are synthesized bacteria by LuxI family protein, and diffused outside and inside the cell. Some AHLs requires active transport through an efflux pump [3]. When intracellular concentration of AHLs increases, LuxR family protein binds AHL and regulates the expression of specific genes [2]. AHL-mediated quorum sensing regulates the various phenotypes such as bioluminescence, motility, pigment production, and biofilm formation [1,2]. In general, AHL-deficient mutants show defects in quorum-sensing regulated gene expression. Therefore, it is expected that disrupting or manipulating the AHL signals could inhibit the expression of quorum-sensing regulated phenotypes such as virulence and infection of host cells [4].

The cooling water systems are used to remove heat generated in the various industries such as chemical and petrochemical, electric power generating stations, and paper mills [5]. Biofouling, which is accumulation of microorganisms on the wet surfaces, is one of the serious problems in the cooling water systems. Biofouling of the cooling water systems causes blocking of condenser pipes and the heat exchanger tubes [5]. Chlorine is one of the most used chemicals for controlling biofouling in the industrial cooling water systems. However, to inhibit biofouling with effect, it is necessary for continuous or intermittent chlorination in the industrial cooling water systems. In the case of the membrane bioreactor (MBR) wastewater treatment systems, quorum sensing is associated with the formation of a biofouling layer on the membrane surface [6]. Quorum-quenching technique has been investigated as an approach to controlling biofouling in MBR wastewater treatment systems [6,7]. However, there is no research example that describes the relationship between biofouling of the cooling water systems and quorum sensing. In a previous study, the AHL synthase gene homolog were found within 26% of the 265 completed Proteobacterial genomes [8]. It was revealed that the majority of the bacterial community in an industrial cooling water system was Proteobacteria [5]. From these facts, there is a high possibility that AHL-mediated quorum sensing affects the development of biofouling in the cooling water system. To our knowledge, the AHL-producing bacterial community in the industrial cooling water system has not been examined. In this study, we screened for AHL-producing bacteria from the industrial cooling water systems, and identified AHL structures produced by them.

## 2. Experimental Section 

### 2.1. Isolation of Bacteria from Industrial Cooling Water Systems

The cooling water samples were collected from the industrial cooling water systems of factory A, B, C, D, and E. The water samples were serially diluted in sterilized distilled water and spread on 1/5-diluted trypticase soy broth (TSB) agar plate (Becton, Dickinson and Company). The plates were incubated in the dark at 30 °C for 72 h. Thereafter, bacterial colonies were randomly selected from among those that grew on the 1/5-diluted TSB plate. Colonies from each sample were randomly selected and transferred onto a new 1/5-diluted TSB agar medium.

### 2.2. Screening of AHL-Producing Bacteria

AHLs were detected by two AHL biosensors, *Chromobacterium violaceum* CV026 and VIR07. Strain CV026 and VIR07 respond to short-chain and long-chain AHLs respectively, by producing the purple pigment violacein [9,10]. The bacterial isolates were inoculated onto a 1/5-diluted TSB agar medium prepared in the 96-well plates. Strain CV026 and VIR07 were inoculated onto the lower right or left positions of the same well. The bacterial colonies were incubated at 30 °C for 24 h. AHL-producing colonies, which induced the production of purple pigments of the AHL reporter strains, were selected.

### 2.3. Identification of AHL-Producing Bacteria

Chromosomal DNA of AHL-producing bacteria was extracted using DNeasy Blood and Tissue Kit (QIAGEN). To identify the bacterial species of AHL-producing bacteria, the 16S rRNA gene-encoding regions were amplified by PCR with Blend Taq plus DNA polymerase (Toyobo, Osaka, Japan) and the previously described universal primers, 27f (5′-AGA GTT TGA TCM TGG CTC AG-3′) and 1525r (5′-AGG AGG TGW TCC ARC C-3′) [11]. After electrophoretic separation, 16S rRNA fragments were purified from agarose by using MagExtractor PCR and Gel Clean up (Toyobo). Sequencing reactions were performed with the BigDye Terminator, ver. 3.1 and Applied Biosystems 3500 Series Genetic Analyzer (Applied Biosystems). Closest type-strain 16S rRNA gene relatives to each clone sequence were determined using the RDP II sequence match tool [12]. A phylogenetic tree by the neighbor-joining method was constructed by Molecular Evolutionary Genetic Analysis (MEGA) [13].

### 2.4. Extraction of AHLs

AHL-producing bacterial strains were inoculated into 4 mL of 1/5-diluted TSB medium and incubated at 30 °C for 18 h with shaking. Two milliliter of full-grown cultures of bacterial strains were transferred into 200 mL of fresh 1/5-diluted TSB medium and incubated at 30 °C for 24 h with shaking. After cultivation, cells were removed by centrifugation at 10,000 × g for 5 min. The culture supernatant was concentrated by evaporation using a rotary evaporator. The concentrated supernatant was extracted with three times its volume of ethyl acetate in a separatory funnel. The extract was evaporated to dryness using a rotary evaporator and was then dissolved in 500 μL of dimethylsulfoxide. To check the presence of AHL, full-grown culture of CV026 or VIR07 was mixed with LB agar medium. Eight-millimeter paper disks, which were applied AHL samples, were placed on LB agar medium containing CV026 or VIR07. After overnight incubation at 30 °C, the appearance of purple pigment was determined as the presence of AHLs.

### 2.5. AHL Identification by Liquid Chromatography-Mass Spectrometry (LC-MS)

Identification of AHL were performed using a triple quadrupole/linear ion trap instrument (LIT) (QTRAP5500; AB Sciex) with an electrospray ionization (ESI) source and coupled to an UHPLC system (Nexera X2; Shimadzu). Chromatographic separation was achieved on a C18 column (Kinetex F5, ϕ 2.1 × 150 mm, 2.6 μm; Phenomenex). For the analysis of AHL, the elution of the samples was carried out using acetonitrile (solvent A) and water (solvent B), both of which contained 0.1% (vol/vol) acetic acid the following gradient (vol/vol): initially 10% A, rising to 50% A at 1.5 min, followed by a 7 min gradient to 99% A. Finally, the column was equilibrated for 3 min, using this solvent composition. The column was operated at 30 °C with a flow rate of 0.2 mL/min. MS/MS spectra were recorded in product ion scan mode using LIT. Ion source was maintained at 400 °C with curtain gas at 20, collisional activated dissociation (CAD) gas at 7 psi (12 psi for LIT), ion source gas at 80 psi, and ion source gas2 at 70 psi. Ionspray voltage was set at 5500 V in positive ion mode. Declustering, entrance, and collision cell exit potentials were maintained at 15 V. Injection volume (2 μL) and the settings used was as described previously. Precursor ion scanning experiments were performed in positive ion mode for analysis, where Q1 was set to scan a mass range of *m/z* 80 to 500 Da and Q3 was monitor for the product ion that indicates presence of lactone ring at *m/z* 102. AHLs were analyzed by LC-MS/MS with MS/MS and precursor ion scanning experiments by identification from synthetic AHLs standards. AHL standards, *N*-hexanoyl-l-homoserine lactone (C6-HSL), *N*-(3-oxohexanoyl)-l-homoserine lactone (3-oxo-C6-HSL), *N*-(3-oxooctanoyl)-l-homoserine lactone (3-oxo-C8-HSL), *N*-decanoyl-l-homoserine lactone (C10-HSL), and *N*-(3-oxodecanoyl)-l-homoserine lactone (3-oxo-C10-HSL), were synthesized using a previously described method [14].

### 2.6. Nucleotide Sequence Accession Numbers

The nucleotide sequences of 16S rRNA were deposited in the DDBJ/ENA/GenBank database with the following accession numbers, LC102389-LC102396.

## 3. Results and Discussion

### 3.1. Isolation and Identification of AHL-Producing Bacteria

A total of 192 bacterial strains were isolated from 5 different factories and examined for the further study. As the results of screening of AHL-producing bacteria using *C. violaceum* CV026 and VIR07, 7 bacterial strains showed AHL-producing activity. 16S rRNA gene were amplified by PCR and sequenced for identification of bacterial species (Table 1). AHL-producing strain D10, which was isolated from factory B, was assigned to the *Aeromonas hydrophila*. Two AHL-producing strains F6 and F13, which were isolated from factory C, were assigned to the *A. hydrophila* and *Lysobacter brunescens*, respectively. Four AHL-producing isolates, which were isolated from factory D, were assigned to the *Methylobacterium oryzae* (strain D7 and F9) and *Bosea massiliensis* (strain E9 and F12). 

**Table 1:** Identification and characterization of *N*-acylhomoserine lactone (AHL)-producing strains on 96-well plates. Induction of AHL-responsible violacein production by CV026 or VIR07 is represented by the number of plus signs; +, low induction; ++, high induction; -, no induction.

Strain	Source	Related Type Strain	Identity	CV026	VIR07
D10	Factory B	*Aeromonas hydrophila*	100%	++	+
F6	Factory C	*Aeromonas hydrophila*	100%	++	+
F13	Factory C	*Lysobacter brunescens*	96.5%	-	++
D7	Factory E	*Methylobacterium oryzae*	99.5%	+	+
E9	Factory E	*Bosea massiliensis*	100%	++	+
F9	Factory E	*Methylobacterium oryzae*	99.5%	-	+
F12	Factory E	*Bosea massiliensis*	100%	++	+

### 3.2. Phylogenetic Analysis of AHL-Producing Isolates

**Figure 1:**
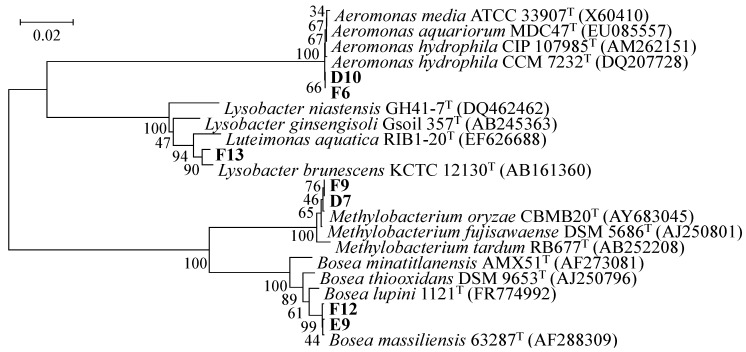
Phylogenetic tree of 16S rRNA gene sequences from AHL-producing isolates. The bacterial isolates in this study were shown in bold style. DDBJ/ENA/GenBank accession numbers are given in parentheses. The phylogenetic tree was constructed by the neighbor-joining method with the ClustalW program of MEGA. The percentage of replicate trees in which the associated taxa clustered together in the bootstrap test (1000 replicates) are shown next to the branches. The scale bar represents 0.02 substitutions per nucleotide position.

Phylogenetic analysis was carried out to examine the relationships of AHL-producing isolates (Figure 1). *A. hydrophila*, which is close to strain D10 and F6, is found in freshwater environments and well-known AHL-producing bacteria [15]. It has been reported that AHL-producing *Aeromonas* strains were isolated from activated sludge [11,15]. It has been reported that *Aeromonas* strains mainly produced *N*-butyryl-l-homoserine lactone (C4-HSL) and *N*-hexanoyl-l-homoserine lactone (C6-HSL) [11]. From the results of AHL-production assay, it was assumed that the *A. hydrophila* D10 and F6 can produce the short chain AHLs such as C4-HSL and C6-HSL as well as reported AHL-producing *Aeromonas* species. *Methylobacterium* species were often isolated from the tap water and well-known AHL-producing bacteria [16,17]. *Methylobacterium* have been reported to exhibit higher stress-tolerance than the other species found in biofilms, particularly under conditions of extreme nutrient limitation [18]. On the other hand, *Lysobacter* sp. and *Bosea massiliensis* have not been reported as AHL-producing bacteria. This is the first report of an AHL-producing bacterial strain belonging to the genus *Lysobacter* and *Bosea*.

### 3.3. Analytical Identification of AHL Molecules

**Figure 2:**
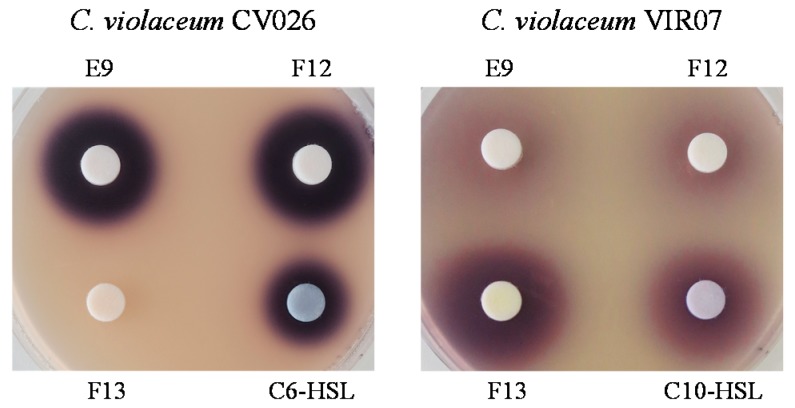
Identification of AHLs produced by *B. massiliensis* E9 and F12, and *Lysobacter* sp. F13. The presence of AHLs was visualized as pigments produced by *C. violaceum* CV026 and VIR07. *N*-hexanoyl-l-homoserine lactone (C6-HSL) and C10-HSL were used for positive control.

**Figure 3:**
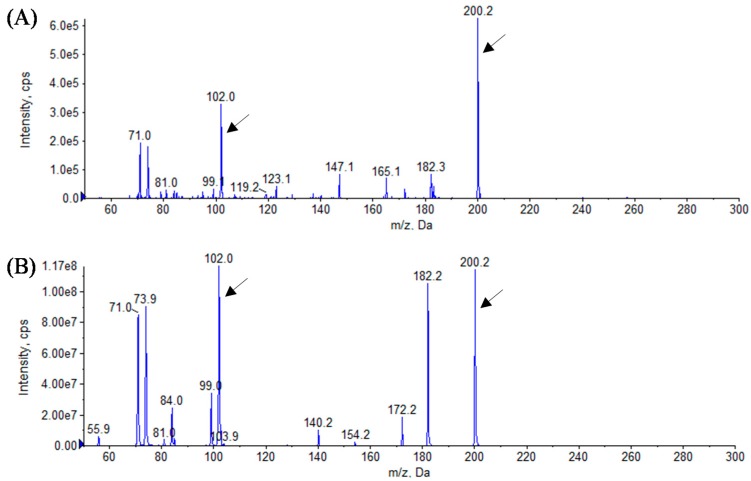
Mass spectra of AHLs extracted from the cell-free supernatant of *B. massiliensis* E9 (**A**) and C6-HSL standard (**B**). All corresponding peaks for respective C6-HSL (*m*/*z* 200) along with the product ion peaks (*m/z* 102) are marked by arrows.

**Figure 4:**
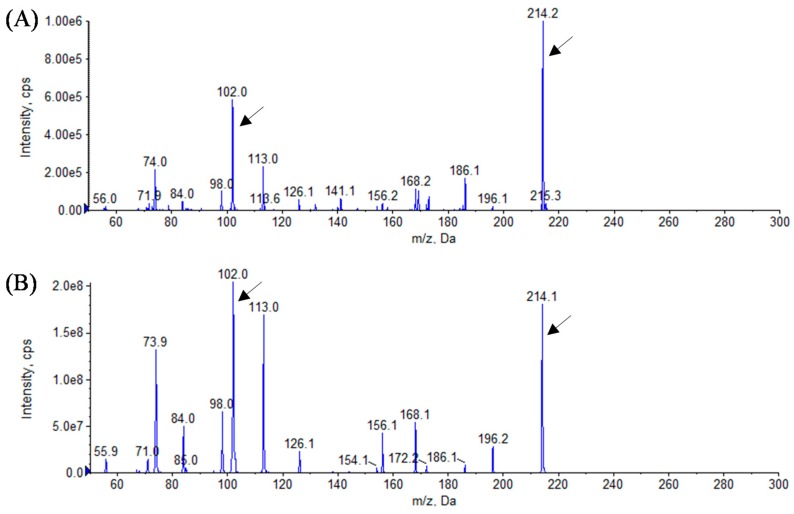
Mass spectra of AHLs extracted from the cell-free supernatant of *B. massiliensis* E9 (**A**) and 3-oxo-C6-HSL standard (**B**). All corresponding peaks for respective 3-oxo-C6-HSL (*m*/*z* 214) along with the product ion peaks (*m/z* 102) are marked by arrows.

**Figure 5:**
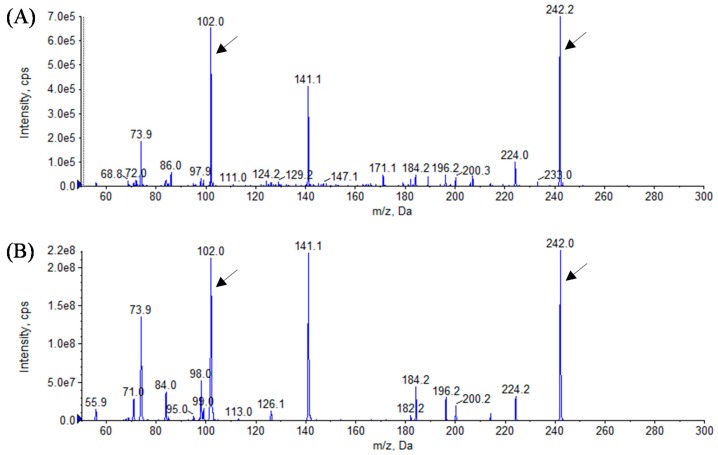
Mass spectra of AHLs extracted from the cell-free supernatant of *B. massiliensis* E9 (**A**) and 3-oxo-C8-HSL standard (**B**). All corresponding peaks for respective 3-oxo-C8-HSL (*m*/*z* 242) along with the product ion peaks (*m/z* 102) are marked by arrows.

**Figure 6:**
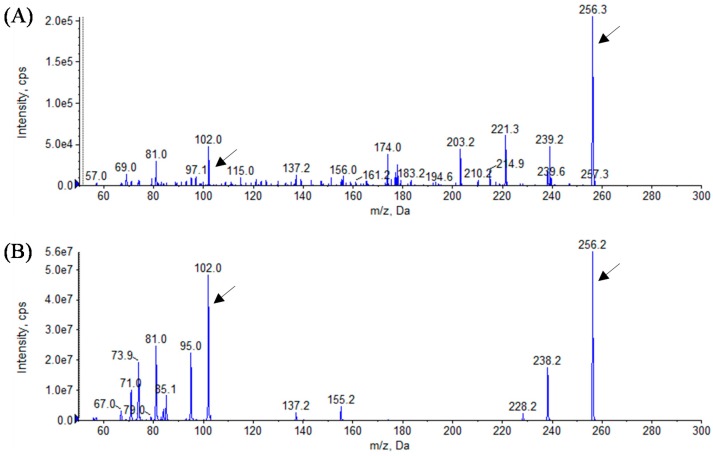
Mass spectra of AHLs extracted from the cell-free supernatant of *Lysobacter* sp. F13 (**A**) and C10-HSL standard (**B**). All corresponding peaks for respective C10-HSL (*m*/*z* 256) along with the product ion peaks (*m/z* 102) are marked by arrows.

**Figure 7:**
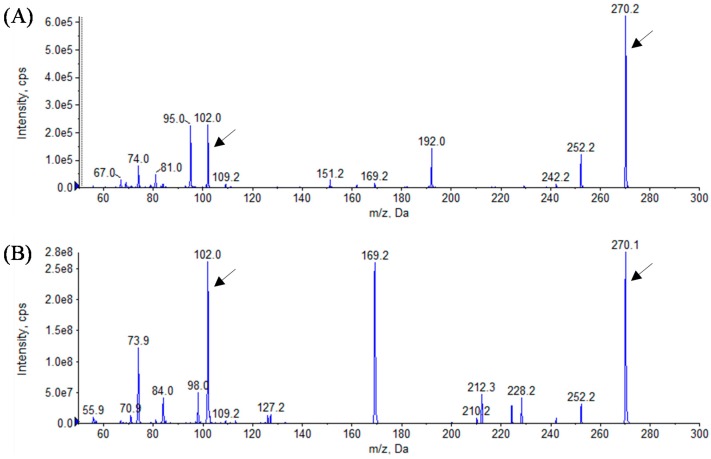
Mass spectra of AHLs extracted from the cell-free supernatant of *Lysobacter* sp. F13 (**A**) and 3-oxo-C10-HSL standard (**B**). All corresponding peaks for respective 3-oxo-C10-HSL (*m*/*z* 270) along with the product ion peaks (*m/z* 102) are marked by arrows.

In this study, we identified the AHL profiles of *B. massiliensis* E9 and F12, and *Lysobacter* sp. F13, which have not been reported as AHL-producing species. To identify the structure of AHL produced by these strains, AHL samples were extracted from the culture supernatants of these strains. The presence of AHLs was checked by LB agar plates containing *C. violaceum* CV026 and VIR07. As the results, AHL extracts from *B. massiliensis* and *Lysobacter* sp. strongly stimulated *C. violaceum* CV026 and VIR07, respectively (Figure 2). These results corresponded to those of 96-well plate assay in Table 1. HPLC fractionation followed by MS analysis of the AHL extracts of these strains were performed to identify the types of AHLs. The presence of C6-HSL, 3-oxo-C6-HSL, and 3-oxo-C8-HSL was identified in the AHL extracts of *B. massiliensis* E9 (Figure 3, Figure 4 and Figure 5). The profile of the AHL extracts of strain F12 completely corresponded to that of strain E9. The presence of C10-HSL and 3-oxo-C10-HSL was identified in the AHL extracts of *Lysobacter* sp. F13 (Figure 6 and Figure 7). Two short-chain AHLs, C6-HSL and 3-oxo-C6-HSL, can stimulate the production of violacein by *C. violaceum* CV026 [9]. Three long chain AHLs, 3-oxo-C8-HSL, C10-HSL, and 3-oxo-C10-HSL, can stimulate the production of violacein by *C. violaceum* VIR07 [10]. AHL profiles of these three strains support the results of the AHL-production assay shown in Table 1 and Figure 2.

## 4. Conclusions/Outlook

In this study, it was found that there were various AHL-producing bacteria in the industrial cooling water system. *Bosea* have been reported as opine degraders in rhizosphere and an amoeba-resisting bacteria [19]. Interestingly, *Bosea thiooxidans* isolated from the tobacco rhizosphere exhibited the ability to inactivate AHL [20]. *Lysobacter* have been reported as ubiquitous beneficial bacteria emerging as novel biocontrol agents and a new potential source of anti-infectives [21]. Another quorum-sensing system, which was mediated by the diffusible signal factor (DSF), has been identified from *Lysobacter enzymogenes* [21]. However, there is no example of AHL-producing bacteria belonging to genus *Bosea* and *Lysobacter*. Many gram-negative bacteria use AHL-mediated quorum sensing to regulate the secretion of sticky extracellular polysaccharide (EPS), which is cause for biofouling [22]. AHL-degrading techniques have been investigated as an approach to controlling biofouling in membrane bioreactor (MBR) wastewater treatment systems [7,23]. Future studies of the relationship between EPS formation and AHL-mediated quorum sensing in cooling water isolates may provide useful information for the control of biofouling.
